# Treatment of Acne Vulgaris by Topical Spironolactone Solution Compared With Clindamycin Solution

**DOI:** 10.7759/cureus.17606

**Published:** 2021-08-31

**Authors:** Adil Noaimi, Shatha R Al-Saadi

**Affiliations:** 1 Department of Dermatology and Venereology, College of Medicine, Baghdad University, Baghdad, IRQ; 2 Dermatology Centre, Baghdad Teaching Hospital, Baghdad, IRQ

**Keywords:** acne vulgaris, spironolactone, clindamycin, comedone, solution

## Abstract

Background: Acne vulgaris is a common skin problem that is encountered in daily clinical work, affecting mostly the adolescent and young adult age group. Many topical therapies have been used in the treatment of mild to moderate types of acne vulgaris. However, none of these modalities is uniformly effective; furthermore, acne vulgaris is also associated with relapse and many topical side effects.

Objective: To compare the effectiveness and side effects of topical 2% spironolactone solution and 1.5% clindamycin solution in the treatment of mild to moderate acne vulgaris.

Material and methods: This was a single-blinded therapeutic clinical comparative study conducted at the Dermatology Center at Medical City in Baghdad, Iraq, from April 2019 to March 2020. Sixty-eight patients with mild to moderate acne vulgaris on the face were included. All sociodemographic data related to the disease were recorded for each patient. Patients were divided into two groups according to the type of therapy: group A (35 patients) used 2% spironolactone solution and group B(33 patients) used 1.5% clindamycin solution. All cases in both groups were instructed to use the solutions twice a day for 12 weeks in the same manner. Patients were seen every two weeks to evaluate the response to therapy and to report any topical side effects; then, follow-up was carried out for one month after cessation of therapy to evaluate relapse.

Results: Spironolactone solution significantly decreased comedone count (p < 0.0001), while the clindamycin solution had no effect on comedones. Although spironolactone was slower than clindamycin solution in reaching the maximum therapeutic effect, the reduction in papules was comparable to that of clindamycin, but it exhibited a greater reduction of pustules (p > 0.05) and the Acne Severity Index (ASI; p > 0.05). Patients in the spironolactone group were more satisfied than those in the clindamycin group. Only minimal local side effects were reported in both groups that did not require cessation of therapy.

Conclusion: Spironolactone solution is an effective and well-tolerated topical treatment for mild to moderate acne vulgaris and is superior to clindamycin solution.

## Introduction

Acne vulgaris is a chronic inflammatory disease of the pilosebaceous follicles, characterised by comedones, papules, pustules, nodules, and often scars. It is one of the most common skin diseases, particularly in adolescents and young adults [1].

Many processes within the pilosebaceous unit are involved in the pathogenesis of acne, including excessive sebum production and changes in sebum fatty acid composition, alterations in the hormone microenvironment, follicular hyperkeratinisation, induction of inflammation, and disturbances in the innate and adaptive immune responses. These will result in abnormal function of the pilosebaceous unit, leading to the development of microcomedones, and then to comedones and inflammatory lesions. *Propionibacterium acnes* can also induce and maintain the inflammatory phase of acne [2-4].

There are many topical therapies that target different steps in its pathogenesis. Some of these therapies include keratolytics like salicylic acid and sulphur, comedolytics like topical retinoids, and antibacterial and anti-inflammatory agents like clindamycin, erythromycin, dapsone, azelaic acid, and benzoyl peroxide [5].

Systemic spironolactone, a synthetic 17-lactone steroid, acts as a non-selective mineralocorticoid receptor antagonist for both progesterone and androgen receptors. It also exhibits anti-androgen effects through the inhibition of the cytochrome p450 system, inhibition of 5 alpha-reductase activity, and an increase in the hepatic synthesis of sex hormone-binding globulin [6]. Topical spironolactone has been used as a gel formulation of 5% concentration, with studies revealing different cure rates [7,8].

Clindamycin is an antibiotic that acts by binding irreversibly to a site on the 50S subunit of the bacterial ribosome, thus inhibiting the translocation steps of protein synthesis [9]. Its beneficial effects in acne are thought to be due to both antimicrobial and anti-inflammatory effects; the latter includes inhibition of the production of polymorphonuclear chemotactic factor, lipase, and neutrophil chemotactic factor by *P. acnes* [9].

Topical clindamycin with different formulations and concentrations has been a standard therapeutic acne treatment for many years [10]. However, it should be noted that the susceptibility of propionibacteria to clindamycin appears to have fallen over the last 42 years [10].

Because of the multifactorial pathogenesis of acne, a search for a further new treatment is going; therefore, the present work was arranged to assess the effectiveness and side effects of topical 2% spironolactone solution and 1.5% clindamycin solution in the treatment of mild to moderate acne vulgaris.

## Materials and methods

This was a single-blinded comparative therapeutic clinical trial carried out at the Dermatology Centre, Medical City, Baghdad, Iraq, between April 2019 and March 2020.

A total of 73 patients were enrolled in this study. Patients with mild to moderate acne vulgaris on the face, including non-inflammatory acne (open and closed comedones) and inflammatory papules and pustules, were enrolled. Exclusion criteria were severe and nodulocystic acne, pregnancy, and lactation. Also excluded, patients who were using topical or systemic medications for acne in the last two months prior to starting therapy, and those who had received laser resurfacing, chemical peeling, mesotherapy, or any similar facial interventions.

Formal consent was obtained from each patient before starting the trial of treatment. A full explanation of the nature, course, treatment, prognosis, and complications of the disease was provided, as well as the target of the present work regarding the drug, its efficacy, side effects, and the method and duration of the treatment and follow-up.

Ethical approval was obtained from the Scientific Council of Dermatology and Venereology of the Iraqi Board for Medical Specialisations. Colour photographs of each patient were taken at each visit. Frontal, right, and left views were taken using a Samsung Galaxy S6 Edge 16-megapixel rear camera in the same place with fixed illumination and distance.

The severity index was recorded for each case according to the following formula:

Acne Severity Index (ASI) = papules + (2 × pustules) + (comedones/2) [11]

Patients were divided into two groups regarding the type of therapy.

In group A, 37 patients were treated with 2% topical spironolactone solution. The solution was prepared as follows: 2 g of spironolactone (ALCTONER, produced by MedicoLabs, Homs Syria, 100 mg) in the form of 20 tablets crushed with a coffee grinder and dissolved in 75 ml rectified spirit and 25 ml distilled water mixed and kept in the hospital in a dark glass container.

In group B, 36 patients were treated with 1.5% topical clindamycin solution. The preparation of the solution was carried out as follows: One and a half grams clindamycin (LanacinR produced by Pharma International Co., Amman, Jordan, 150 mg) in the form of ten capsules was dissolved in 75 ml rectified spirit and 25 ml distilled water mixed and kept in the hospital in a dark glass container. Patients in both groups were instructed to shake the bottle well before use and apply the solution on a gauze pad, which was then compressed on the face in a relatively full-field therapy twice daily for 12 weeks.

Clinical evaluation was done every two weeks till the end of twelve weeks. Then the patients were asked to stop the use of medication to be re-evaluated after one month without any treatment. The assessment was carried out by counting the lesions (i.e., comedones, papules, and pustules), calculating the ASI, watching for any topical or systemic side effects, and evaluating patient satisfaction.

The satisfaction of patients with treatment was classified into: (i) full satisfaction, (ii) partial satisfaction, and (iii) no satisfaction.

Statistical analysis

All data were coded and entered into the computer using Statistical Package for Social Sciences (SPSS) version 26 (IBM Corp., Armonk, NY). Comparison before and after treatment in each group was made by using a paired t-test. A p-value < 0.05 was considered significant. An F-test (ANOVA) was applied to measure the significant differences between and within groups.

## Results

Five (6.9%) patients did not complete the study because of loss of follow-up due to difficulty in their transport (two in group A and three in group B). Sixty-eight patients completed the course of treatment. Table 1 shows the demographic data for the patients in both groups.

**Table 1:** Sociodemographic information of the patients in the study groups.

	Group A (n=35)	Group B (n=33)
Age	18.3 ± 4.6	17.8 ± 3.8
Male	9 (25.7%)	8 (24.2%)
Female	26 (74.3%)	25 (75.8%)

Assessment of the mean ± SD of the comedone, papule, and pustule counts and the ASI at each visit within the groups

The mean ± SD of the comedone, papule, and pustule counts and the ASI at each visit for both groups are shown in Tables 2 and 3.

**Table 2:** Mean ± SD of the comedone, papule, and pustule counts and the ASI of the patients in group A at each visit. ASI: acne severity index.

	Comedones	Papules	Pustules	ASI
First visit	91 ± 64.4	5.7 ± 3.9	4.2 ± 3	60.1 ± 32.3
2 weeks	88.4 ± 63	3.6 ± 3.5	2.3 ± 2.3	52.5 ± 31.1
4 weeks	82.1 ± 61.5	1.8 ± 2.4	1 ± 1.5	43.1 ± 31.1
6 weeks	75 ± 59	0.4 ± 0.9	0.4 ± 1.1	38.6 ± 28.9
8 weeks	68.9 ± 56.8	0 ± 0.3	0 ± 0.3	34.7 ± 28.2
10 weeks	62.7 ± 54.8	0 ± 0.3	0 ± 0.3	31.5 ± 27.3
12 weeks	57 ± 52.9	0 ± 0.3	0 ± 0	28.5 ± 26.4
F-test	196	51	47	136
P-value	0.0001	0.0001	0.0001	0.0001

**Table 3:** Mean ± SD of the comedone, papule, and pustule counts and the ASI of the patients in group B at each visit. ASI: acne severity index.

	Comedones	Papules	Pustules	ASI
First visit	67.6 ± 34.6	6.2 ± 2.6	4.7 ± 2.7	49.5 ± 20.3
2 weeks	67.4 ± 35	2.5 ± 1.7	1.8 ± 1.9	39.9 ± 19.9
4 weeks	67.7 ± 35.5	1 ± 1.4	0.7 ± 1.3	36.3 ± 19.6
6 weeks	66.8 ± 34	0.5 ± 1.3	0.5 ± 1.2	34.9 ± 18.3
8 weeks	66.8 ± 35	0.4 ± 1	0.4 ± 1	34.6 ± 18.9
10 weeks	66.6 ± 35.5	0.1 ± 0.5	0.2 ± 0.6	33.9 ± 18.4
12 weeks	66.7 ± 35	0.1 ± 0.6	0 ± 0.3	33.7 ±18.1
F-test	1	70	53	75
P-value	>0.05	0.0001	0.0001	0.0001

**Figure 1:**
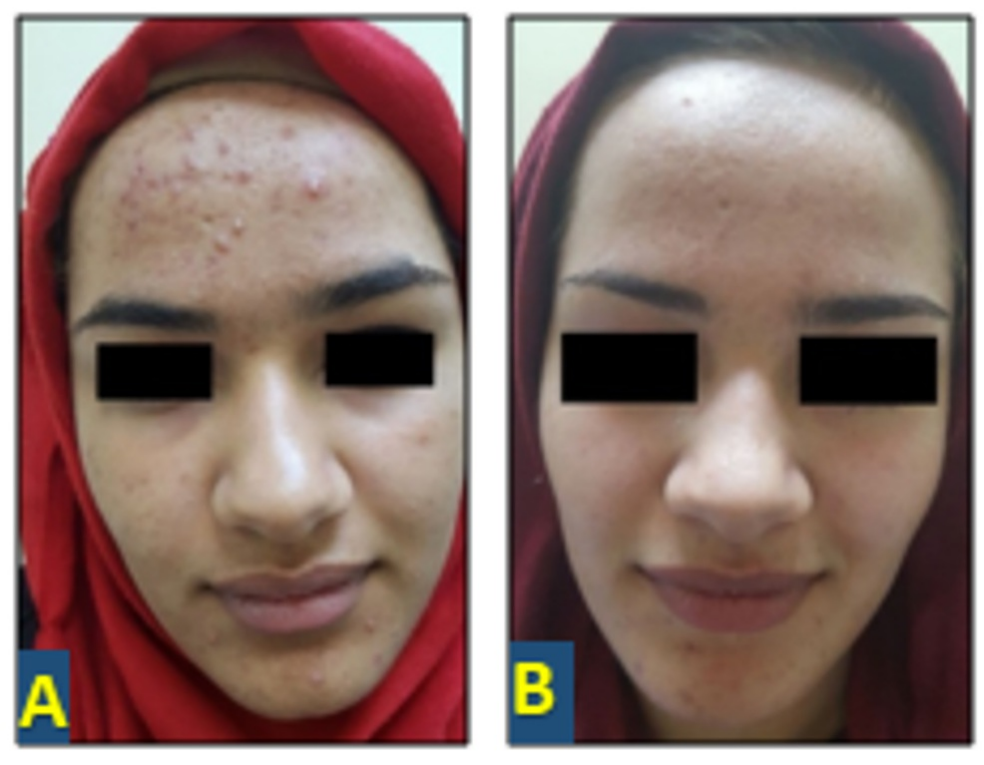
Seventeen-year-old female with moderate acne vulgaris: (A) before treatment and (B) twelve weeks after treatment with 2% topical spironolactone solution.

**Figure 2:**
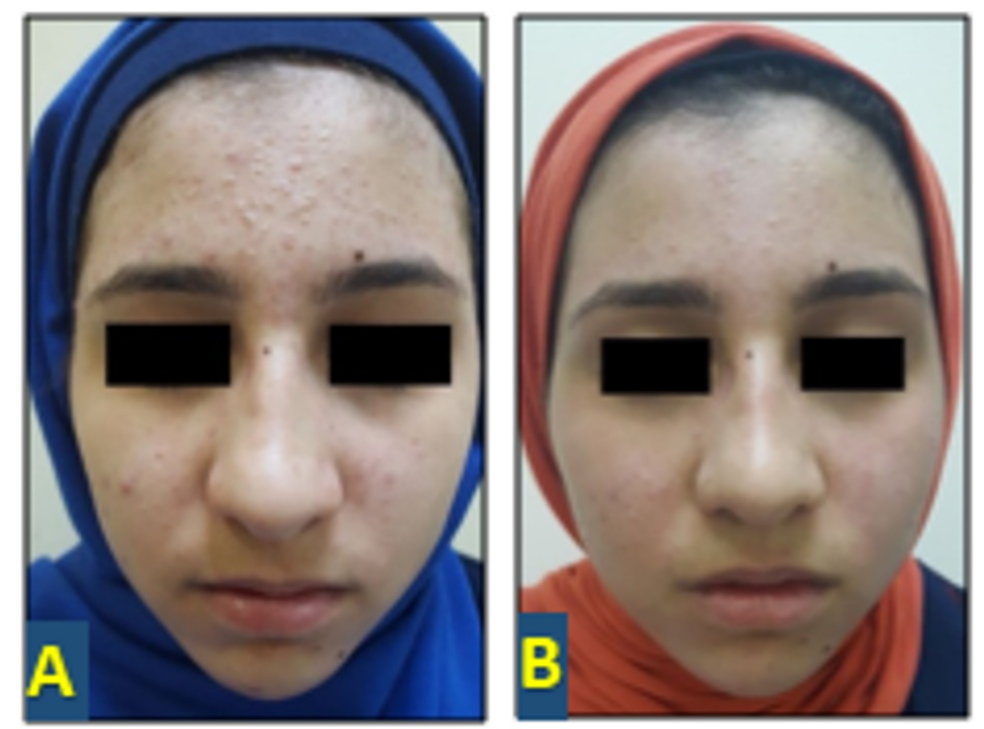
Fourteen-year-old female with mild acne vulgaris: (A) before treatment and (B) 12 weeks after treatment with 1.5% topical clindamycin solution.

At the end of 12 weeks, in group A, the total reduction of comedones was 37% (p < 0.0001, highly significant), that of papules was 94.7% (p < 0.0001, highly significant), that of pustules was 100% (p < 0.0001, highly significant), and that of the ASI was 52.5% (p< 0.0001, highly significant; Figure 1). There was no effect on comedone count in group B; the total present reduction of papules was 98% (p < 0.0001, highly significant), that of pustules was 93% (p<0.0001, highly significant), and that of ASI was 31.8% (p < 0.0001, highly significant; Figure 2).

Assessment of the mean ± SD of ASI four weeks after stopping the treatment (relapse rate) as compared with that at 12 weeks within the groups

In group A, the mean ± SD of ASI at 12 weeks was 28.5 ± 26.4. Four weeks after stopping the treatment, these variables were reduced to 28 ± 25.6 (statistically not significant). In group B, the mean ± SD of the ASI at 12 weeks was 33.7 ± 18.1. Four weeks after stopping the treatment, these variables changed to 33.8 ± 17.9 (statistically not significant).

Comparing the effect between the two groups

**Figure 3:**
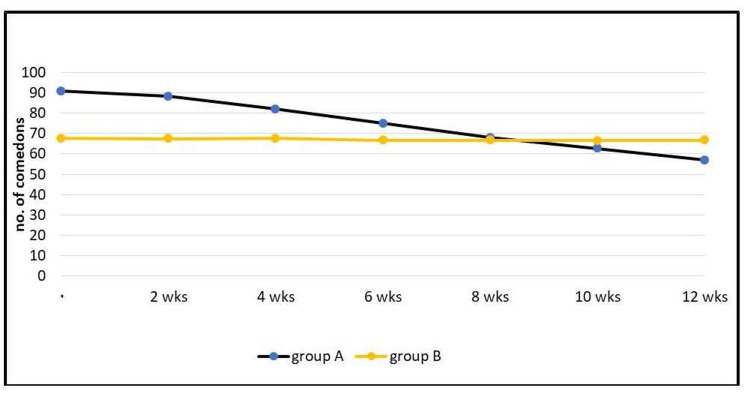
Effect on comedone counts in each group. wks: weeks.

Regarding comedones, in group A, the mean ± SD of the comedone count at the first visit for the patients was 91 ± 64.4; at the last visit (at the 12th week), the mean ± SD was 57 ± 52.9. In group B, the mean ± SD of the comedone count at the first visit for the patients was 67.6 ± 34.6, and at the last visit (at the 12th week), it was 66.7 ± 35. Statistical analysis showed that only group A had a reduction in the comedone count, and this reduction was highly significant (p < 0.0001; Figure 3).

**Figure 4:**
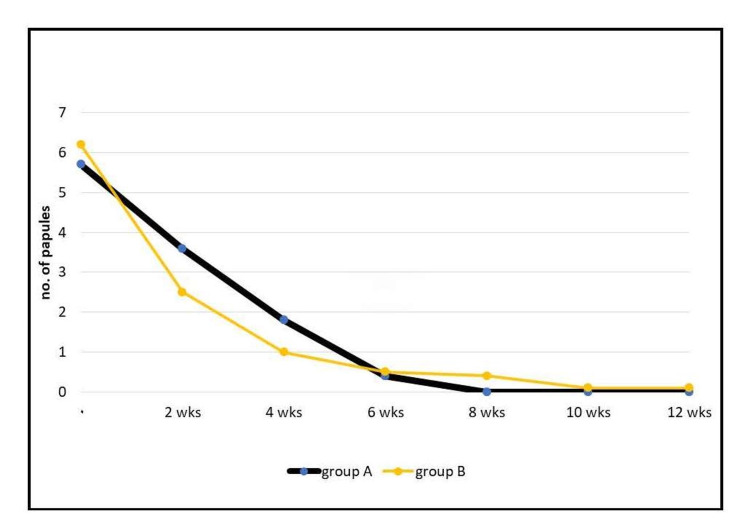
Demonstrating the effect on papules in each group. wks: weeks.

Regarding papules, in group A, the mean ± SD of the papules at the first visit for the patients was 5.7 ± 3.9, and at the last visit (at the 12th week), it was 0 ± 0.3. In group B, the mean ± SD of papules at the first visit for the patients was 6.2 ± 2.6, and at the last visit (at the 12th week), it was 0.1 ± 0.6. Statistical analysis showed that group B had a greater reduction in the number of papules than group A. However, the difference was not statistically significant (p > 0.05; Figure 4).

**Figure 5:**
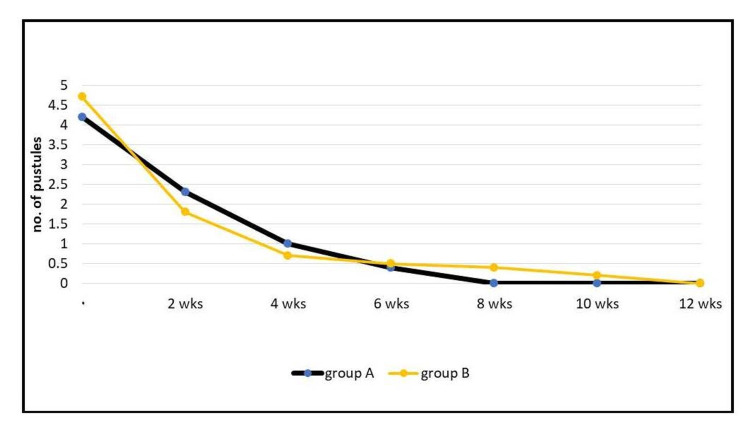
Elucidating the effect on pustules in each group. wks: weeks.

Regarding pustules, in group A, the mean ± SD of the pustule count at the first visit for the patients was 4.2 ± 3, and at the last visit (at 12th week), it was 0 ± 0. In group D, the mean ± SD of the pustule count at the first visit for the patients was 4.7 ± 2.7, and at the last visit (at the 12th week), it was 0 ± 0.3. Statistical analysis showed that the reduction of pustules was greater in group A than in group B. However, the difference between the groups was not statistically significant (p > 0.05; Figure 5).

**Figure 6:**
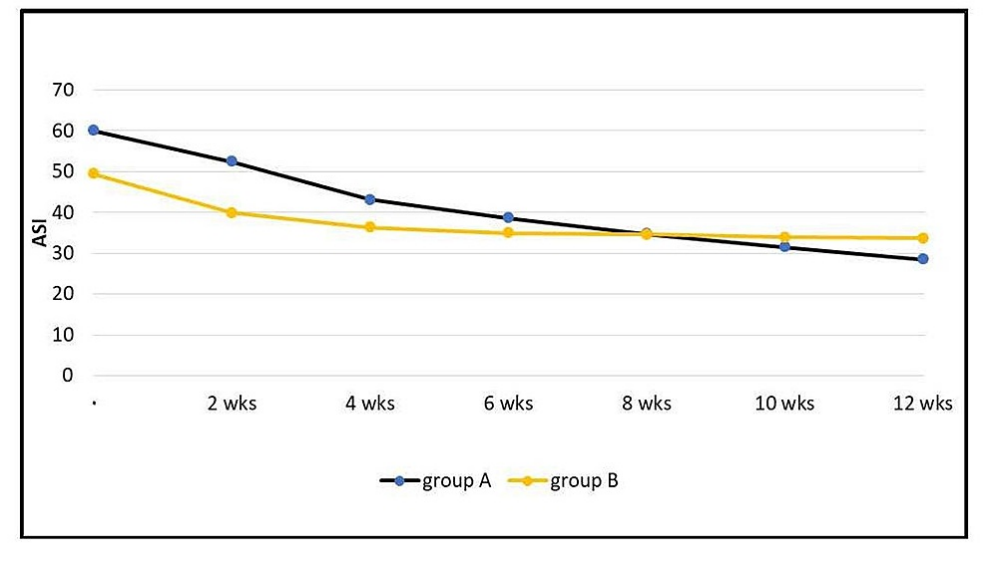
The effect on ASI in each group. wks: weeks, ASI: acne severity index.

Regarding the ASI [1], in group A, the mean ± SD of the ASI at the first visit for the patients was 60.1 ± 32.3, and at the last visit (at the 12th week), it was 28.5 ± 26.4. In group B, the mean ± SD of the ASI at the first visit for the patients was 49.5 ± 20.3, and at the last visit (at the 12th week), it was 33.7 ± 18.1. Statistical analysis showed that group A had a greater reduction in the ASI, and this reduction was higher than that of group B and was also statistically significant (p < 0.05; Figure 6).

Assessment of the side effects

In group A, two (5.7%) patients reported an itching sensation, and four (11.4%) had a burning sensation; in group B, three (9.1%) patients had an itching sensation and six (18.2%) patients had a burning sensation. All the side effects were mild and did not require cessation of the treatment.

Assessment of patients’ satisfaction

In group A, 31 (88.6%) patients were fully satisfied, three (8.6%) patients were partially satisfied, and one (2.9%) patient was not satisfied; in contrast, in group B, 29 (87.9%) patients were fully satisfied, three (9.1%) patients were partially satisfied, and one (3%) patient was not satisfied.

## Discussion

Acne vulgaris is a common dermatological problem that is encountered in routine clinical practice. In spite of numerous local treatments meant to address different steps in the pathogenesis of the disease, none could completely clear up all types of acne lesions (comedones, papules, and pustules). Further, the majority were associated with relapse and many local side effects [10].

For this reason, researchers are still trying to find new remedies that target all or most stages in the pathogenesis and obtain a highly curable rate with minimal local side effects and relapse rates. Therefore, the present work was designed to assess the therapeutic effects of spironolactone in comparison with clindamycin.

In this study, the use of topical spironolactone resulted in a 100% reduction in the number of pustules, a 94.7% reduction in the number of papules, and a 37% reduction in the number of comedones. It also decreased ASI by 52.5%. All these reductions were statistically significant, and a maximum effect was observed after six weeks of treatment. Further, a reduction in the number of comedones continued until the end of the 12th week. None of the patients showed signs of recurrence during the follow-up period.

These results are in accordance with those reported in the studies by Afzali et al. [7], Kalidari et al. [12], and Attwa et al. [8]. However, the reduction in the inflammatory lesions (papules and pustules) was greater in this study, probably because of the use of a solution rather than a gel formulation, in spite of the higher concentration of spironolactone in the gel formulation (5%) versus the solution (2%) in the present work. Another reason could be the longer duration of therapy (12 weeks), which was reported to be eight weeks in other studies. The only topical side effects were itching (5.7%) and burning (11.4%), which were comparable to other studies [7,8,12].

We can conclude that the therapeutic effects of spironolactone, through percutaneous absorption, are probably due to its antiandrogenic action, as it inhibits 5α-reductase activity [13], thus interfering with sebum production and subsequent synthesis of proinflammatory cytokines [14]. In addition, decreased sebum production will lead to a change in the media within the pilosebaceous unit and make it inhospitable for *P. acnes* [13]. This explains why topical spironolactone showed its maximal effect relatively late in this study, yet it has anticomedonal and anti-inflammatory effects that make it look like a topical retinoid, aside from the keratolytic properties of the latter. Moreover, spironolactone is a mineralocorticoid receptor antagonist, and these have been demonstrated to inhibit inflammation by decreasing inflammatory cytokines, regulating ion channel expression and activity, and reducing tissue oedema [15,16].

Clindamycin is an antibiotic that acts by binding to the bacterial 50S ribosomal subunit, thus inhibiting protein synthesis. It is topically utilised in the treatment of acne vulgaris, with contributions from its anti-inflammatory and antibacterial effects [13]. In this study, a 1.5% clindamycin solution prepared by the researcher was used as the gold standard. The result was a statistically significant reduction in inflammatory lesions, including a 98% reduction in the number of papules, a 93% reduction in the number of pustules, and a 31.8% decrease in ASI, which was also statistically significant. A maximum effect was noticed from the fourth week and was maintained until the end of the 12th week. However, the number of comedones was not significantly affected. Most patients did not show any sign of relapse during the follow-up period.

These results were comparable to those of previous studies by Zhang et al. and Alirezaï et al. [17,18], which also showed significant reductions in only the inflammatory lesion count. Topical side effects were itching (5.7%) and burning (18.2%), which were parallel to other studies [19].

## Conclusions

Topical spironolactone solution is superior to clindamycin topical solution because it has multiple therapeutic effects on acne vulgaris lesions, especially a reduction of comedones, with a high cure rate and minimal relapse, despite the fact that clindamycin has antibacterial and anti-inflammatory actions.

So we strongly recommend using topical spironolactone alone or in combination with other antibiotics for the treatment of mild to moderate acne vulgaris.
